# A Huge Primary Congenital Splenic Cyst Diagnosed in a Middle‐Aged Female Patient: A Case Report

**DOI:** 10.1002/ccr3.71006

**Published:** 2025-10-15

**Authors:** Bungech Kouy, Suk‐Kyu Oh, Hour Leng, Chantha Run, Vireak Chhun, Young Don Lee

**Affiliations:** ^1^ Department of Education & Training Hebron Medical Center Phnom Penh Cambodia; ^2^ Department of Surgery Hebron Medical Center Phnom Penh Cambodia; ^3^ Department of Pathology Hebron Medical Center Phnom Penh Cambodia

**Keywords:** non‐parasitic cyst, primary congenital cyst, splenectomy, splenic cyst

## Abstract

Splenic cysts are classified into primary (true) and secondary (pseudo or false) cysts. Primary non‐parasitic congenital splenic cysts are comparatively rare, particularly in the middle‐aged population. We present a rare case of a huge primary congenital splenic cyst, incidentally discovered in a female patient in her mid‐50s. This case report aims to highlight the uncommon presentation of primary congenital splenic cysts in middle age and emphasizes the necessity of considering primary congenital splenic cysts in the differential diagnosis even in patients beyond middle age.

## Graphical abstract

Gross appearance of a splenic cyst showing the smooth external surface (left) and trabeculated inner wall (right).

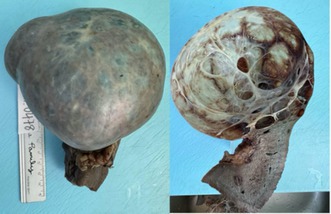

**Summary**

- Primary congenital cysts are typically diagnosed in childhood or adolescence, with cases identified after the age of 50 being extremely rare.
- We report a case of a huge primary congenital cyst, histologically confirmed in a female patient diagnosed in her mid‐50s.

## 1 Introduction

Splenic cysts are uncommon findings in routine medical practice, and large splenic cysts are especially rarely encountered. Although the true prevalence of splenic cysts remains unclear, a review of over 42,000 autopsies suggested an incidence of approximately 0.07% [1, 2, 3]. Splenic cysts are classified into primary cysts, defined by the presence of an epithelial lining, and secondary cysts, which are absent of this lining [1, 4, 5, 6]. Primary cysts can be either parasitic (also known as hydatid cysts, most commonly caused by *Echinococcus granulosus*) or non‐parasitic [7]. Primary non‐parasitic splenic cysts are classified into congenital and neoplastic types, and cysts lined by mesothelial cells were categorized as congenital cysts [4]. Secondary cysts are usually of post‐traumatic origin. Secondary cysts are the most common type overall, and primary cysts are relatively rare.

Small splenic cysts do not cause any noticeable symptoms, and mostly splenic cysts are discovered at a large size or after complications, such as rupture, infection, or hemorrhage, have occurred [1, 6, 8]. Recent advancements in the use of abdominal imaging have led to increased incidental findings of splenic cysts [4, 6, 7].

Primary non‐parasitic congenital cysts are predominantly observed in pediatric and younger adult populations and in middle‐aged adults, the primary congenital cysts remain scarce [8, 9, 10]. We report a case of a 54‐year‐old female with a large primary non‐parasitic congenital splenic cyst.

## 2 Case History/Examination

A 54‐year‐old female Cambodian presented with a 2‐month history of a palpable left‐sided abdominal mass as her sole presented symptom. It was associated with mild abdominal distension but no significant abdominal pain. No gastrointestinal symptoms, including nausea, vomiting, or diarrhea, were observed. Her medical history was notable only for hypertension, with no prior surgical interventions or any trauma to the abdomen. Physical examination revealed no apparent abdominal asymmetry on inspection due to the patient's overweight stature. However, palpation identified a firm, regular‐bordered, large mass in the left upper quadrant, which was non‐tender and without guarding.

## 3 Differential Diagnosis, Investigation, and Treatment

**FIGURE 1:**
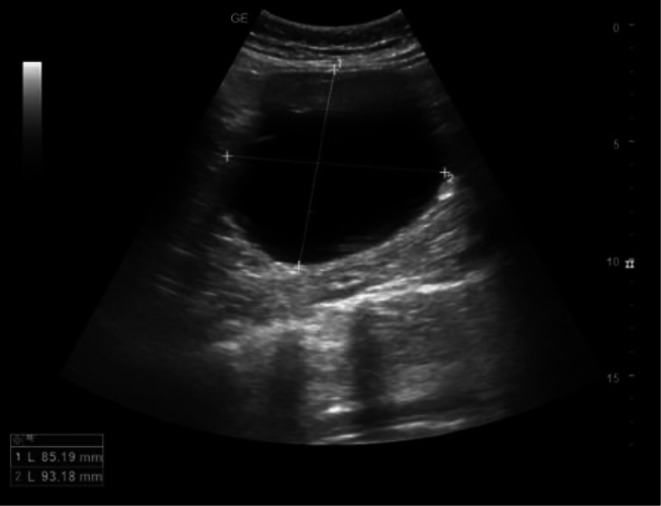
Abdominal ultrasonography revealed a cystic lesion with a well‐defined border located in the left hypochondriac–lumbar region.

**FIGURE 2:**
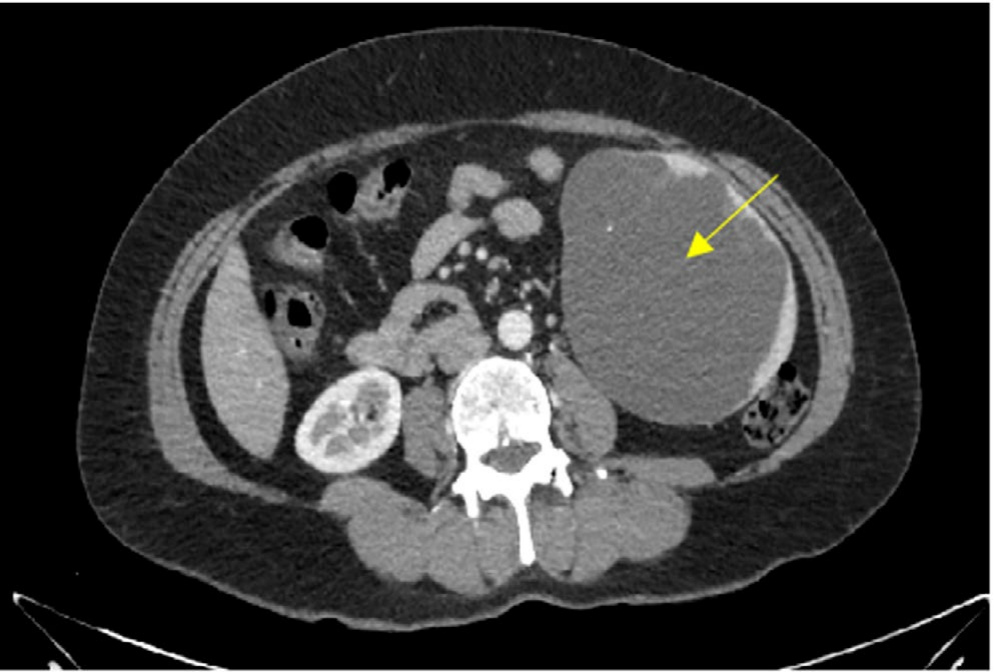
Axial abdominal CT revealed a large cyst (yellow arrow) located at the lower pole of the spleen.

The basic laboratory test results were as follows: hemoglobin, 13.1 g/dL; white blood cell count, 8600/μL; platelet count, 245,000/μL; blood urea nitrogen/creatinine, 29.0/0.9 mg/dL; aspartate aminotransferase/alanine aminotransferase (AST/ALT), 17/18 IU/L; electrolytes (Na^+^–K^+^–Cl^−^), 140–4.2–102 mmol/L; urinalysis, clear; prothrombin time international normalized ratio (PT INR), 1.06; prothrombin time, 11.9 s; activated partial thromboplastin time (aPTT), 29.3 s. Abdominal ultrasonography revealed a round anechoic lesion with a well‐defined margin at the left hypochondriac‐lumbar region with a rough measurement of 85 × 93 mm (Figure 1). Abdominal computerized tomography (CT) revealed a large, well‐defined, thin‐walled homogeneous unilocular cyst located in the inferior pole of the splenic parenchyma. There were no internal septations or rim enhancement, and the diameter of the cyst was approximately 100 × 120 mm (Figure 2).

**FIGURE 3:**
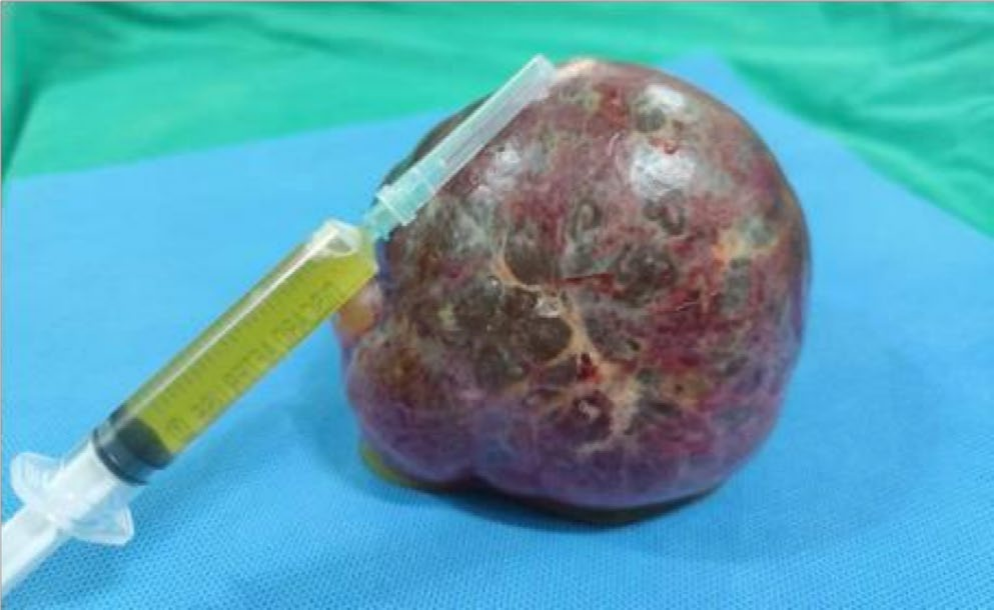
Clear yellowish fluid was aspirated from the splenic cyst after the successful open total splenectomy.

The open splenectomy was performed. The surgical procedure was as follows: An open total splenectomy was performed using a 120 mm upper midline incision under general anesthesia. In the operation findings, a large cyst with a diameter of approximately 100 mm was seen at the inferior pole of the spleen. There were some adhesions in the splenorenal area at the superior pole, and the hilum vessels were smaller than normal. The surgeon carefully separated the splenorenal ligament, gastrosplenic attachment, and vessels at the inferior pole. The splenic artery was then dissected, and the splenic hilum was double ligated. Finally, the spleen was separated from the pancreatic tail. The whole spleen together with the cyst was securely removed without any rupture. A Jackson‐Pratt drain was inserted, and the wound was then closed layer by layer. After successfully removing the whole spleen along with the unruptured cyst, the fluid aspirated from the cyst was clear yellowish (Figure 3). There were no intraoperative difficulties, and no evidence of parasitic infection such as septation or honeycomb pattern in the operation field and pathologic findings. In addition, no postoperative infection or mechanical complications were observed.

## 4 Outcome and Follow‐Up

**FIGURE 4:**
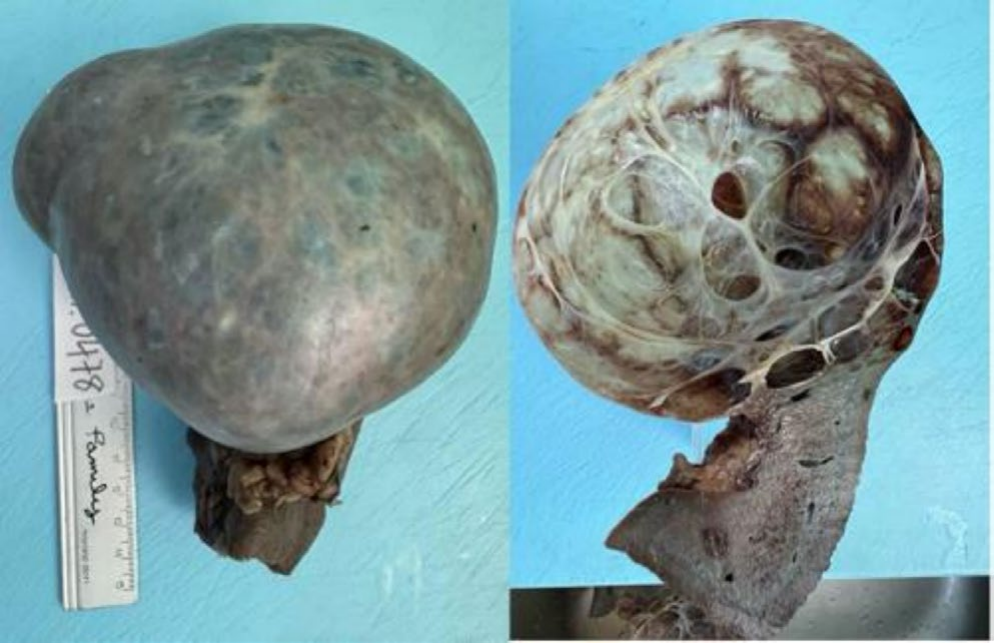
The gross findings of the splenic cyst.

**FIGURE 5:**
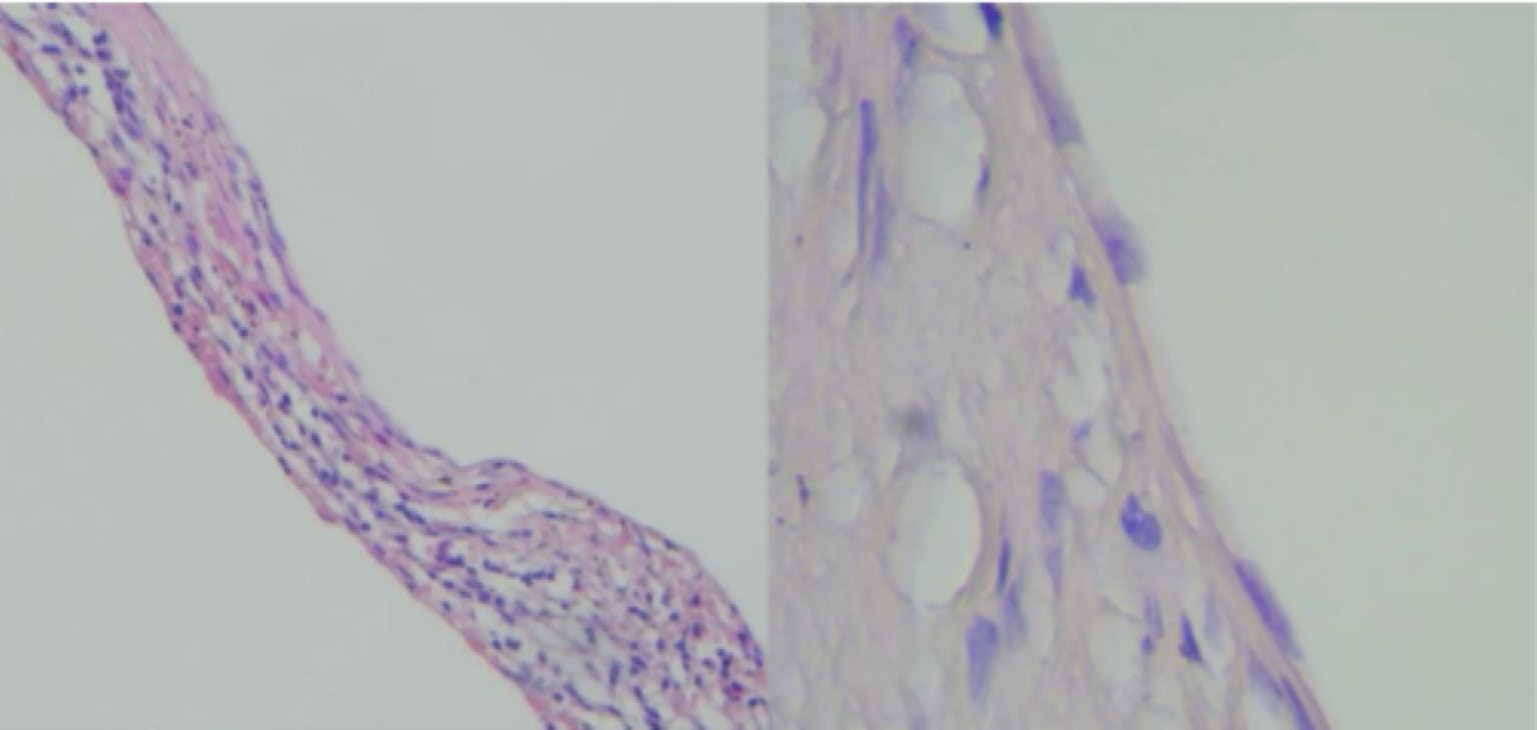
The microscopic findings of the splenic cyst wall lined by a single‐layered flattened or cuboidal epithelial cell (Hematoxylin and Eosin stain, magnification ×40 and ×100).

According to the gross findings, the spleen size was 145 × 120 × 85 mm with a typically well‐defined round‐shaped cyst, which was 100 × 80 mm in diameter. Grossly, the surface of the cyst was smooth and contained clear yellowish fluid. The inner wall of the cyst was grayish‐white and heavily trabeculated (Figure 4). Microscopically, the cyst wall was lined by a single layer of flattened or cuboidal epithelial cells, which were non‐keratinized (Figure 5). There were no atypical cells or evidence of malignancy in the splenic cyst. The splenic parenchyma was unremarkable.

The patient recovered well after surgery and was discharged on the postoperative day 13 after receiving post‐splenectomy vaccination for *Haemophilus influenzae* (VAXIGRIP) and *Streptococcus pneumoniae* (Prevenar 13). The outpatient follow‐up 1 week after the discharge was uneventful.

## 5 Discussion

We present this unique case to underscore two features. First, primary non‐parasitic large splenic cysts are typically detected during adolescence or early adulthood; however, our case is notable for being diagnosed in the mid‐50s, which is an uncommon presentation. Second, despite being diagnosed at a relatively late age, the cyst was not associated with trauma and did not exhibit any neoplastic features; however, it demonstrated characteristics of a congenital cyst, being lined by a single layer of flattened or cuboidal non‐keratinized epithelial cells.

The majority of primary splenic cysts are parasitic, usually caused by *Echinococcus granulosus*, accounting for 60% of all primary splenic cysts [11]. Primary epithelial cysts are relatively rare, accounting for approximately 10% of all splenic cysts [12]. Congenital cysts are true cysts characterized by an epithelial lining, which can be epidermoid, dermoid, or endodermoid in nature. Congenital epidermoid cysts are characterized by an epithelial lining, which may present as squamous, transitional, or mesothelial epithelium [5]. The wall of this subtype is typically fibrotic and exhibits diverse trabecular architecture, while its contents consist of a yellow, proteinaceous fluid [7]. Primary non‐parasitic congenital splenic cysts are typically detected during adolescence or early adulthood, whereas traumatic secondary splenic cysts are often identified at a later age. Among non‐neoplastic, non‐parasitic splenic cysts in both children and adults, a study of 52 patients found that 24 had primary cysts and 28 had post‐traumatic (secondary) cysts. The average age of patients with primary cysts was 17.9 years (range: 7–32), while the average age of patients with secondary cysts was 30 years (range: 15–62) [13]. Up to 80% of nonparasitic splenic cysts are diagnosed in patients under the age of 20, supporting a congenital etiology [14].

According to a literature review that collected data from 1989 to 2008, among a total of 166 pediatric patients with splenic cysts, 82% were congenital cysts [15]. In a multicenter study conducted from 1981 to 2005, 60% of the 50 patients had congenital cysts [16]. In a single‐center study conducted from 1993 to 2011, 81% of the 21 patients, or 17 patients, had congenital cysts [17].

However, congenital cysts are rarely found in adults. In a study conducted in Iran over 24 years, 16 adult splenic cyst patients were identified, of which 11 had parasitic cysts, 1 had a pseudocyst, and only 4 had non‐parasitic primary congenital cysts [11]. A study analyzing 15 non‐parasitic splenic cysts in adult patients who underwent surgery at a single institution between 1989 and 2001 found that 6 had primary cysts, and 9 had secondary cysts [18].

In our case, interestingly, despite being a remarkably large splenic cyst (100 × 80 mm in diameter), it was detected at an unusually late age. The causes for the late detection are presumed to be twofold: (1) the cyst located in the lower pole caused minimal compressive symptoms on the surrounding organs, (2) the difficulty in accessing routine ultrasound screening due to the healthcare situation in Cambodia. We conducted a literature review of splenic cysts larger than 10 cm found in adults aged 18 and older since 2000 (Table 1). The average age of the 26 adults with splenic cysts was 30.1 years. Most of the patients had symptoms, and the cysts were located in the upper pole in 17 patients, the middle pole in 5 patients, and the lower pole in 1 patient. Compared to existing literature, the lack of significant symptoms and the late discovery at the age of 50 in our patient can be attributed to the cyst's location in the lower pole, which likely did not exert pressure on other organs.

**TABLE 1:** Characteristics of congenital primary splenic cysts larger than 100 mm since 2000.

No.	References	Sex	Age	Size (mm)	Pathologic finding	Symptom	Location
1	Morgenstern (2002) [5]	F	24	270	Mesothelial	LUQ discomfort	Upper pole
2	Morgenstern (2002) [5]	F	40	250	Mesothelial	LUQ discomfort palpable mass	Upper pole
3	Morgenstern (2002) [5]	F	27	120	Epidermoid	LUQ discomfort	Lower pole
4	Morgenstern (2002) [5]	M	34	200	Epidermoid	LUQ discomfort palpable mass	Upper pole
5	Morgenstern (2002) [5]	M	18	140	Transitional	Abdominal pain, palpable mass	Upper pole
6	Morgenstern (2002) [5]	M	32	140	Mesothelial	LUQ discomfort	Middle
7	Morgenstern (2002) [5]	M	44	150	Transitional	LUQ discomfort	Upper pole
8	Morgenstern (2002) [5]	F	34	210	Transitional	LUQ discomfort	Upper pole
9	Morgenstern (2002) [5]	F	30	170	Epidermoid	LUQ discomfort	Middle
10	Morgenstern (2002) [5]	M	29	170	Epidermoid	LUQ discomfort palpable mass	Upper pole
11	Morgenstern (2002) [5]	F	43	200	Epidermoid	LUQ discomfort palpable mass	Upper pole
12	Morgenstern (2002) [5]	F	31	120	Mesothelial	Palpable mass	Upper pole
13	Morgenstern (2002) [5]	F	29	120	Epidermoid	Abdominal pain	Upper pole
14	Morgenstern (2002) [5]	F	31	100	Mesothelial	Palpable mass	Upper pole
15	Morgenstern (2002) [5]	M	30	160	Epidermoid	LUQ discomfort	Upper pole
16	Avital (2003) [19]	F	32	200	Epithelial	Abdominal pain	Upper pole
17	Geraghty (2009) [3]	F	38	100	Epithelial	Abdominal pain	Upper pole
18	Dan (2010) [20]	F	25	180	Epidermoid	Abdominal pain	Upper pole
19	Golmohammadzadeh (2016) [11]	F	19	120	Epithelial	Abdominal pain	Not revealed
20	Golmohammadzadeh (2016) [11]	F	16	210	Epithelial	Abdominal pain	Not revealed
21	Golmohammadzadeh (2016) [11]	F	21	150	Epithelial	Abdominal pain	Not revealed
22	Vuyyuru (2017) [21]	F	51	110	Epidermoid	Abdominal pain	Middle
23	Res (2019) [22]	F	46	11	Epidermoid	Abdominal pain	Middle
24	Coulier (2020) [23]	M	19	21	Epidermoid	Abdominal pain	Upper pole
25	Termos (2020) [6]	F	22	20	Epithelial	Abdominal pain	Upper pole
26	Hammouda (2022) [2]	M	18	10	Epithelial	Abdominal pain	Middle

Despite being a remarkably large cyst discovered at a late age, histologically, it demonstrated features consistent with a primary congenital splenic cyst lined with flattened or cuboidal non‐keratinized epithelial cells, and with an interior exhibiting trabeculation. Based on a review of several references, it is exceedingly rare for a primary congenital cyst to be discovered in a person in their 50s.

## 6 Conclusion

Primary epithelial cysts are relatively rare, accounting for approximately 10% of all splenic cysts. In addition, a splenic cyst exceeding 100 mm in size being discovered in the age of 50s is exceedingly rare. We present a unique case of a primary congenital epithelial cyst exceeding 100 mm in size, which was discovered asymptomatically in a patient over the age of 50.

## Author Contributions

**Bungech Kouy:** writing – original draft. **Suk‐Kyu Oh:** conceptualization, data curation, validation. **Vireak Chhun:** data curation, resources. **Hour Leng:** methodology, resources. **Chantha Run:** methodology, resources. **Young Don Lee:** methodology, resources, supervision.

## Consent

Written informed consent was obtained from the patient to publish this report in accordance with the journal's patient consent policy.

## Conflicts of Interest

The authors declare no conflicts of interest.

## Data Availability

Data sharing not applicable—no new data generated.
